# Dental hypersensitivity in individuals with cleft lip and palate: Origin and therapies

**DOI:** 10.34172/joddd.2021.008

**Published:** 2021-02-13

**Authors:** Viviane Da Silva Siqueira, Aury Elianny Sanchez Castillo, Jose Francisco Mateo-Castillo, Lidiane De Castro Pinto, Daniela Garib, Cláudia Ramos Pinheiro

**Affiliations:** ^1^Dentistry Department, Hospital for Rehabilitation of Craniofacial Anomalies, Universidade de São Paulo (HRAC/USP), Bauru, São Paulo, Brazil; ^2^Postgraduate Center for Dentistry, CPO Uningá, Bauru, São Paulo, Brazil

**Keywords:** Cleft palate, Dental pulp, Dentin desensitizing agents, Dentin sensitivity, Endodontics, Oral hygiene

## Abstract

**Background.** Dental hypersensitivity is due to the exposure of the dentin layer after wear of enamel or cementum, exposing the dentinal tubules and the nerve endings of odontoblasts within these tubules. The present study aimed to assess the factors related to dental hypersensitivity in individuals with cleft lip and palate and the most common therapy received.

**Methods.** The medical records of 536 patients with cleft lip and/or palate (281 males, 255 females) with a mean age of 18 were analyzed in a single center. The inclusion criterion was patients reporting dental hypersensitivity from May 2015 to October 2019. The origin of dental hypersensitivity was evaluated considering orthodontic movement, periodontal diseases, and reversible and irreversible pulpitis. The therapy indicated by the dental professionals for dental hypersensitivity were recorded. Descriptive statistics were performed.

**Results.** Of 61 teeth with dental hypersensitivity, 10 were attributed to orthodontic movement, 21 to periodontal problems, 27 to reversible pulpitis, and three to irreversible pulpitis. The most used therapies were the application of fluoride varnish and prophylaxis, dentifrice indication for dental sensitivity, free gingival grafts, pulpectomy, desensitizing agent application, conservative endodontic treatment (direct pulp protection), and restoration of non-carious cervical lesions.

**Conclusion.** Reversible pulpitis was the most prevalent etiologic factor of dental hypersensitivity in individuals with cleft lip and palate. Dentifrices for dental sensitivity and fluoride varnish application were frequently recommended.

## Introduction

Orofacial clefts are identified as congenital defects that occur during intrauterine life, precisely during the 8th week (cleft lip and alveolar flange) and the 12th week gestational age, when the palatine cleft occurs.^1^ When the hygiene of these individuals is poor, they are susceptible to dental caries and all the harm it causes to the oral environment. When these patients are not assisted by a dentist, and interventions are not performed, the bacteria and their byproducts reach the pulp, and the tooth in question requires endodontic intervention.^2^ The hydrodynamic theory is the most widely accepted hypothesis, and according to this theory, the basis for the transmission of sensation is the movement of fluids in the dentinal tubules.^3^ It is suggested that dental hypersensitivity would be an inflammatory response in the pulp. However, few studies are available on pulp reactions in hypersensitive dentin.^4^

Fagundes-De-Souza et al^4^ evaluated pulp inflammation histologically, investigating this hypothesis through an *in vivo* model to simulate erosion to expose dentinal tubules so that the pulp response could be verified. Sixteen Wistar rats were fed with a sucrose-free commercial diet for 12 hours, while the food was removed for the remainder of the day, and the animals received mineral water or a lemon-based sucrose-free liquid to drink according to the group they belonged to. Eight animals consumed soda to induce hypersensitivity, while the other eight animals received mineral water (control group). After 6 weeks, histological evaluation of the control and experimental groups revealed no pulp inflammation process and the presence of inflammatory cells, such as lymphocytes, plasma cells, eosinophils, and macrophages. In addition, there was no swelling or dilated and congested blood vessels. This animal model showed that dental hypersensitivity does not trigger an inflammatory response of the dental pulp.^4^

It has been reported that periodontal disease might be present, but painlessly or with few symptoms; therefore, patients do not know how to respond to this condition. Midwood et al^5^ investigated patient awareness of periodontal health, dental hypersensitivity, and dental wear, and their impact on the oral health quality of life in patients in southwest England, where 814 adult patients answered an oral health questionnaire and then underwent clinical examinations. The participants exhibited good oral hygiene practices and a low prevalence of periodontitis. For all the conditions evaluated, self-reported data and clinical indices were observed. The results showed strong and weak associations for dental hypersensitivity and tooth wear, respectively, confirming the negative impact of periodontal disease and dental hypersensitivity, related to gingival recession, even in healthy gingiva and patients not susceptible to periodontitis.^5,6^

Orthodontic treatment is essential in rehabilitating cleft lip and/or palate patients who have undergone primary surgeries and who will undergo secondary surgeries. Thus, orthodontic therapy is planned in different stages, one of them before bone graft and the other after a bone graft.^7^ According to Rana et al,^8^ there is a correlation between the severity and extent of gingival recession due to orthodontic treatment that might be associated with dental hypersensitivity. This author suggests that orthodontic movement in some situations causes gingival recession, creating an environment that predisposes this condition, especially if the teeth are repositioned, which is a common clinical treatment procedure in patients with cleft lip and palate.^8^

The present study aimed to assess the factors related to dental hypersensitivity in individuals with cleft lip and palate and the most common therapy received.

## Methods

Medical records (n = 536) of individuals who underwent treatment at the dental center in the last five years were analyzed. Inclusion criteria consisted of patients with any type of cleft lip and palate, with a mean age of 18 in a single center, who had undergone alveolar bone graft surgery and were under orthodontic treatment. The data referring to the causes of dental hypersensitivity and the therapeutic conduct of the professionals’ choice were analyzed and quantified, taking into account the notes made in the medical records by the professional in charge of the care. In addition, at no time the patients’ identities were disclosed. The data were tabulated through descriptive statistics of percentage analysis and graphics.

## Results

**Figure 1:**
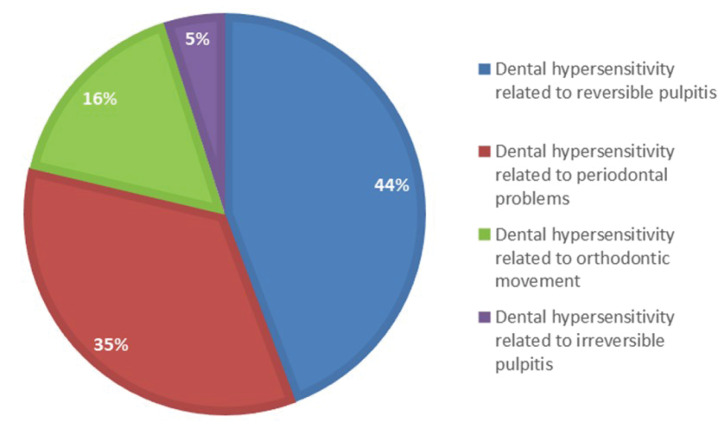
Percentages of dental hypersensitivity according to etiologic factors: individuals with orthodontic movement-related dental hypersensitivity; dental hypersensitivity related to periodontal problems; related to reversible pulpitis-related dental hypersensitivity and individuals with irreversible pulpitis-related dental hypersensitivity.

A total of 536 medical records were analyzed, of which 61 individuals had some type of dental hypersensitivity. Thus, dental hypersensitivity related to reversible pulpitis exhibited a percentage of 44%, the percentage related to periodontal problems was 35%, with 16% related to orthodontic movement and 5% related to irreversible pulpitis (Figure 1).

Different types of treatments were verified to solve the painful symptoms caused by dental hypersensitivity, as follows: application of fluoride varnish and prophylaxis (26%), dentifrice indication for tooth sensitivity (26%), desensitizing agent application (22%), free gingival graft (10%), pulpectomy (10%), conservative endodontic treatment (with direct pulp protection) and restoration of non-carious cervical lesions (3%).

## Discussion

The present study aimed to identify the etiologic factors of tooth hypersensitivity through the analysis of medical records, obtaining a percentage of 44% for reversible pulpitis (Figure 1) as the most prevalent etiologic factor in individuals with cleft lip and/or palate treated at the Hospital Rehabilitation of Craniofacial Anomalies (HRAC/USP). Dental hypersensitivity is due to the exposure of the dentin layer after the wear of the enamel or cementum layer, exposing the dentinal tubules and nerve endings of the odontoblasts within these tubules. This exposure will increase the hydraulic conductivity of the affected dentin, and, in response, the patient reports painful symptoms. Odontoblasts and dental pulp cells can be sensitized by mechanical, chemical, or thermal stimuli.^9^

When performing tests that assess the pain response, caution should be exercised to establish a differential diagnosis between dentin hypersensitivity and pulp inflammation because similar symptoms might appear in both clinical situations. Tooth sensitivity is triggered by cold, heat, and air, while in pulp inflammation, the pain has clinical characteristics of being spontaneous, long-lasting, not always localized, resulting in excitation of the C, myelinated, and thermoreceptor fibers, generally related to an irreversible inflammatory process. The response, unlike the pain of hypersensitivity, is well located and acute and disappears after the stimuli are removed. As a result of the excitement over the myelinated delta-A and thermoreceptor fibers, the inflammatory response is reversible.^10^ The most used pulp sensitivity tests are generally electrical and thermal tests (cold or hot).^11^ Dental hypersensitivity can also occur due to the lack of oral hygiene performed by individuals and by dentists manipulating their teeth to control periodontal disease.^12^

Dental caries leading to reversible pulpitis makes the conservative treatment of the pulp necessary using direct or indirect capping, resulting in the formation of dentin bridges.^13^

Meticulous control of the plaque is an important factor in reducing hypersensitivity. Hypersensitivity to dentin due to periodontal treatment presents an initial symptom of acute pain caused by external stimuli applied to exposed dentinal tubules.^14^

The present study, through retrospective analysis in individuals with cleft lip and/or palate, showed a significant percentage of reversible pulpitis as an etiologic factor of dental hypersensitivity since it is directly related to the hygiene of these disabled individuals, making them susceptible to dental caries and all the damage they cause to the oral environment. The results showed a percentage of 35% for periodontal problems as a significant etiologic factor because hypersensitivity might occur due to poor oral hygiene. The etiology of gingival recession is multifactorial, including excessive and inadequate brushing, destructive periodontal disease, poor dental positioning, alveolar bone dehiscence, thin and delicate root surface, high muscle adhesion and frenal traction, occlusal trauma, and other iatrogenic factors.^12-14^ The present investigation suggests that teeth previously traumatized by injury to periodontal tissues are more susceptible to dental hypersensitivity.

Orthodontic forces induce the release of chemical mediators, modifying blood circulation and cellular metabolism, finally causing pulp inflammation.^15^ These processes, depending on their duration and strength, result in interferences with the pulp’s metabolic activity, causing cellular damage or defense reactions. High levels of AST (aspartate aminotransferase) have been detected in the inflamed pulp tissue and gingival fluids related to orthodontic treatment. Enzymes such as AST are released only in the extracellular environment in cases of cell death in which the applied force can alter the physiological state of the pulp elements, causing hypersensitivity and increased levels of AST. If not excessive, they can only cause transient changes.^16^ According to the medical records in the present study, in 16% of individuals, orthodontic movements were responsible for tooth hypersensitivity.

Bauss et al^17^ carried out a retrospective study in orthodontically treated patients to investigate the influence of tooth extrusion and the risk of pulp necrosis for this reason. The results showed that moving teeth by orthodontic forces can affect the blood supply to the dental pulp. Dental pulp inflammation (pulpitis) is a process that involves various neuronal and vascular reactions. Neuropeptides are actively involved in homeostatic regulation, both under normal conditions and during pulp inflammation, controlling their blood flow and regulating them after inflammation and repair processes. Therefore, in the present study, irreversible pulpitis was considered an etiologic factor for dental hypersensitivity with the lowest percentage, with only 5% of the reports found.^10^

Regarding the treatments used in cases of dental hypersensitivity, we can classify them into the following groups: anti-inflammatory agents (corticosteroids), protein precipitants (formaldehyde, silver nitrate, strontium chloride hexahydrate), tubule blockers (calcium hydroxide, potassium nitrate, sodium fluoride), tubule sealants (adhesive resins), and others. However, the results of these therapies are unpredictable.^18^

**Figure 2:**
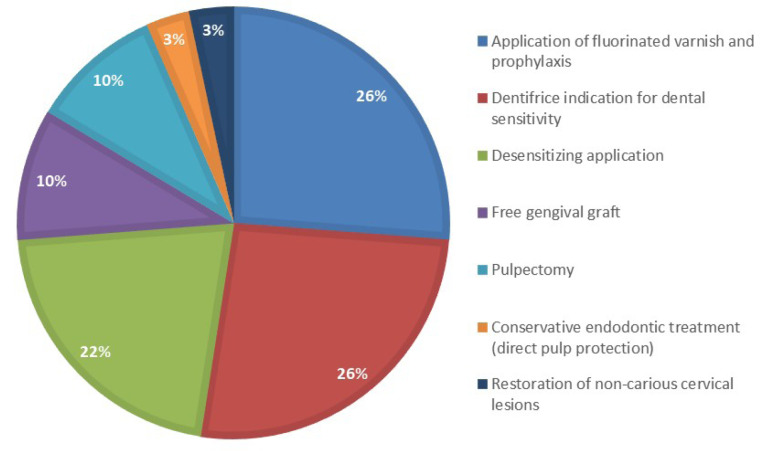
Percentages of treatments performed on individuals with dental hypersensitivity.

The treatments commonly performed in the present study are described in Figure 2. These results show a percentage of 26% for the fluoride varnish application associated with prophylaxis and 26% for toothpastes for tooth sensitivity. The treatment of choice for dental hypersensitivity is the use of home care products, such as toothpastes. Toothpastes are important for cleaning teeth and serve as carriers of active ingredients for protection.

In this sense, toothpastes might have special claims associated with their action under specific conditions, such as in the treatment of tooth hypersensitivity (desensitizing creams) or to protect the tooth surface against tooth erosion.^19^ Its active ingredients obliterate the dentinal tubules or desensitize nerves; they can also form a resistant layer on the tooth surface. Therefore, they can prevent dentin hypersensitivity by preventing dental erosion. When it comes to fluoride varnish, the main factor is fluoride, whose protective effect against dental erosion is generally attributed to the formation of a layer similar to calcium fluoride.^20^ Observed at higher concentrations, they were associated with a lower percentage of a successful response.

Concerning treatment by applying a desensitizer, we obtained a percentage of 22%. These treatments lead to the blockage of nerve impulses caused by stimuli. The desensitization mechanism is based on preventing the generation of action potentials in the nerve axons of dental hypersensitive tissue. This is achieved by preventing axon depolarization by increasing the action and concentration of extracellular potassium since under normal conditions, there is a high concentration of extracellular sodium and a high concentration of intracellular potassium. Performing this procedure prevents the change of electrical charges on both axon sides, with the consequent membrane-stabilizing effect of this nerve cell.^9-21^

We also identified a percentage of 10% for the treatment of dental hypersensitivity through free gingival graft. Several surgical techniques are used to treat gingival recessions; the free epithelial graft or free gingival graft are known to prepare supraperiosteal dissection to remove the epithelium and connective tissue. Among the techniques mentioned, connective tissue free graft has important advantages compared to the post-surgical epithelial free gingival graft; the laterally dislocated flap is a highly anticipated technique, whose main advantage is that the patient’ feels pain because the donor area is adjacent to the recipient surface (avoiding the need for two surgical areas). Coronally displaced flap is indicated for the treatment of localized gingival recessions when there is no edentulous area.^21^

The results of the present study demonstrated that 10% of treatments performed for dental hypersensitivity resulted in radical endodontic treatment. There were some cases with severe dental hypersensitivity, leading to endodontic intervention (pulpectomy).^16^ Caries is the etiologic factor commonly related to the need for endodontic treatment.^2^ However, another frequent indication of endodontic intervention is the prosthetic preparations when the patient’s dental hypersensitivity cannot be resolved with conservative treatments.

da Silva Costa et al^22^ reported that success in treating dental hypersensitivity would depend on the dentist’s scientific knowledge to ensure accurate identification of etiologic factors and the correct selection of materials to desensitize and restore, that is, the correct clinical intervention. In the present study, we obtained a percentage of 3% for the restoration of non-carious cervical lesions as the treatment of choice for dental hypersensitivity and 3% for conservative treatment of the pulp (direct pulp capping).^22^

Most cleft patients need some type of rehabilitation treatment, indicating the significance of our work in determining the etiologic factors of dental hypersensitivity and the most commonly used treatments, considering the importance of maintaining their teeth.

## Conclusion

According to the present study findings, reversible pulpitis was the most prevalent etiologic factor in individuals with cleft lip and palate with dental hypersensitivity. Dentifrices for sensitivity and fluoride varnish application were the most frequently performed treatments.

## Authors’ Contributions

VDSS initiated, conceptualized, and supervised the research work. AESC, JFMC, and LDCP prepared samples and performed the study with the collaboration of CRP, LDCP, and VDSS. All authors have contributed to analyzing the data and writing the manuscript.

## Acknowledgments

The authors would like to thank the Hospital for the Rehabilitation of Craniofacial Anomalies - University of São Paulo, for the authorization of this research, as well as the patients who contributed to the study.

## Funding

The work has no funding.

## Competing Interests

We declare no conflict of interest.

## Ethics Approval

The study was approved by the Human Research Ethics Committee under the code 07762819.5.0000.5441 of the Hospital for Rehabilitation of Craniofacial Anomalies - University of São Paulo (HRAC/USP).
